# How I do it: pediatric lateral-type posterior fossa ependymoma resection

**DOI:** 10.1007/s00701-026-06960-x

**Published:** 2026-06-25

**Authors:** Katarína Horčičáková, Jakub Táborský, Kateřina Trková, Vladimír Beneš

**Affiliations:** 1 Department of Neurosurgery, Second Faculty of Medicine, Charles University and Motol and Homolka University Hospital, Prague, Czech Republic; 2 Center for Pediatric Neuro-Oncology, Motol and Homolka University Hospital, Prague, Czech Republic; 3 Department of Pediatric Haematology and Oncology, Second Faculty of Medicine, Charles University and Motol and Homolka University Hospital, Prague, Czech Republic

**Keywords:** Ependymoma, Pediatric, Surgical technique, Cranial nerves

## Abstract

**Background:**

Lateral-type ependymomas, or cerebellopontine ependymomas, of the posterior fossa are rare tumors extending into the cerebellopontine angle, encasing important neurovascular structures, presenting as a particular surgical challenge.

**Method:**

We performed a radical resection of lateral-type ependymoma in a 2-year-old patient with anamnesis of torticollis and vomiting. Resection of the tumor was preceded by insertion of external ventricular drainage.

**Conclusion:**

Optimal treatment improving prognosis requires achieving gross total resection of the tumor.

**Supplementary Information:**

The online version contains supplementary material available at https://doi.org/10.1007/s00701-026-06960-x.

## Case summary

**Fig. 1:**
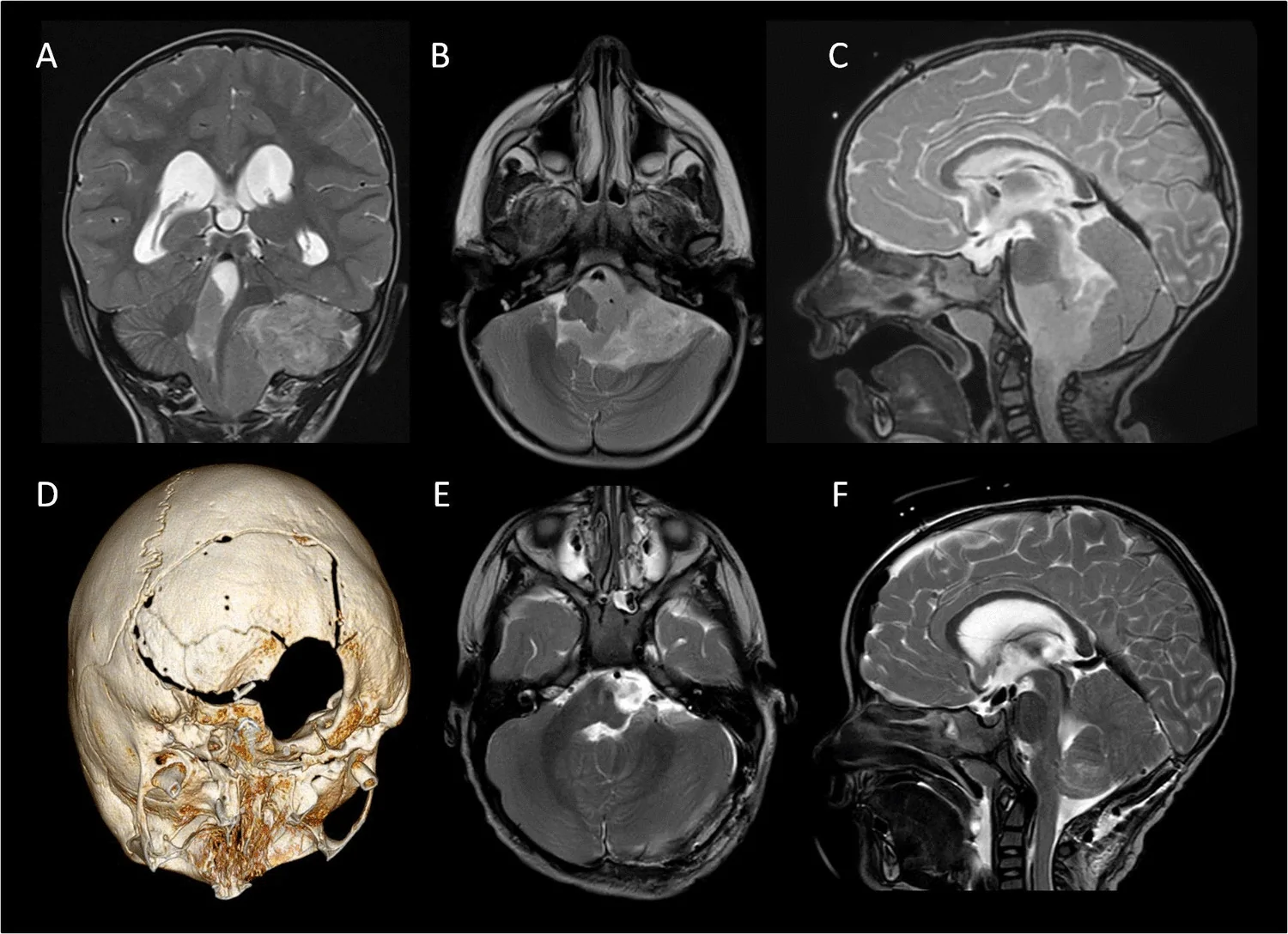
Preoperative coronal (**A**), axial (**B**) and sagittal (**C**) T2- weighted MRI images. Note the tumor extending from the foramen of Luschka into the fourth ventricle, cerebellopontine angle and in front of the brainstem. **D **— computer tomography reconstruction shows the extent of the craniotomy. Corresponding postoperative MRI in axial (**E**) and sagittal (**F**) planes confirming gross total resection

A 2-year-old boy with a lateral-type ependymoma of the posterior fossa (Fig. 1ABC) presented with torticollis and vomiting. He underwent gross total resection (GTR) of the tumor with insertion of ventricular drainage two days prior. Histopathological and genetic evaluations confirmed anaplastic ependymoma CNS WHO grade 3, PFA group. Postoperatively, transient swallowing dysfunction and left-sided hemiparesis occurred and resolved completely at three-month follow-up.

## Relevant surgical anatomy

Understanding the distorted microanatomy of the lateral-type ependymoma is of paramount importance. Unlike midline fourth ventricular ependymomas, lateral-type ependymomas typically originate from the lateral recess of the fourth ventricle, from the foramen of Luschka or lateral aspect of the brainstem. As they expand, they extend laterally through the foramen of Luschka into the cerebello-pontine angle (CPA) and may reach the prepontine cistern anterior to the brainstem. During this process neurovascular structures of the posterior fossa are encased into the folds of the tumor, the brainstem is rotated and cranial nerves elongated. Important anatomical landmarks include vertebral arteries (VA) as they enter the intracranial space, their junction and anterior spinal artery. One of VAs is usually readily identifiable below the tumor [4].

Cranial nerves (CN) are best appreciated laterally as they exit the posterior fossa through their respective neuroforamina and their course can then be traced proximally through the tumor towards their origin. Following the spinal root of n. XI allows early identification of the jugular foramen.

## Description of the technique

Under general anesthesia, the patient was positioned prone, followed by registration of neuronavigation and initiation of intraoperative neurophysiological monitoring (IOM). A left-sided far-lateral approach extending across the midline was combined with a retrosigmoid approach and C1 laminectomy using hockey-stick skin incision (Fig. 1D). Durotomy was performed along the venous sinuses in order to allow maximal exposure.

The tumor was initially gradually dissected along its border and separated from the cerebellar hemispheres, tonsil and vermis. The arachnoid between the tonsils was sharply dissected and the tonsil was elevated thus exposing foramen of Luschka already enlarged by the tumor. The floor of the fourth ventricle was identified and carefully protected. The origin of the tumor was identified in the foramen of Luschka and addressed by a combination of microsurgical dissection and bipolar cautery. Care was taken not to disturb the brainstem pia mater in the area of tumor origin.

Upper cervical rootlets as well as spinal rootlets of n.XI were identified; the left VA was exposed after lifting folds of the tumor extending into the upper cervical spinal canal. The dentate ligament was cut at this stage. The VA was followed to help identify the posterior inferior cerebellar artery (PICA), which was incorporated into the tumor. A clip was applied, and after approximately ten minutes without deterioration on IOM, the vessel was sacrificed. Tumor folds were carefully dissected and were found to contain encased neurovascular structures. CNs were identified at their respective foramina and traced within the folds toward their origin from the brainstem. Through alternating progressive tumor debulking and arachnoidal dissection along the neurovascular structures, the tumor was gradually removed via surgical corridors created between the cranial nerves and vessels of the posterior fossa. The left VA was followed to the vertebrobasilar junction, and the contralateral anterior-inferior cerebellar artery was identified. While preserving the arachnoid layers, the tumor was dissected off the anterior surface of the brainstem. This strategy allowed preservation of the lower cranial nerves, the VII-VIII complex, and the abducens nerve. The trigeminal nerve was displaced but not encased by the tumor. A large tumor portion attached to the cerebellum was removed after dissection along its border, another large portion was removed from the fourth ventricle via the foramen of Luschka; in these regions, and the tumor was only loosely attached, thus achieving GTR (Fig. 1EF).

The tumor was soft and highly vascular, and intraoperative histopathological examination confirmed the suspected diagnosis of ependymoma.

The surgical field was continuously irrigated by papaverin to optimize cranial nerve perfusion, and hemostasis was achieved. Dural and wound closure was performed in a standard fashion. IOM remained stable throughout the procedure, with only cranial nerve stimulation responses observed.

## Indications

The current standard of care for pediatric patients with posterior fossa ependymomas consists of maximal safe surgical resection followed by adjuvant focal radiotherapy. Achieving GTR represents the most significant modifiable prognostic factor for survival, as even a minimal residual tumor substantially reduces the probability of long-term survival [4].

Postoperative MRI should be performed within 48 h after surgery to accurately assess the extent of resection. Second-look surgery is strongly recommended when postoperative MRI reveals subtotal resection.

## Limitations

The primary limitation to radical resection of lateral-type ependymomas is the tumor’s aggressive growth pattern, which involves encasement of vital neurovascular structures. This complexity is further exacerbated by the tumor’s tendency to rotate and elevate the brainstem, profoundly distorting normal posterior fossa anatomical landmarks, making surgery exceptionally challenging [4].

Patients diagnosed with lateral-type ependymomas are frequently infants or very young children. Their limited circulating blood volume makes even moderate intraoperative blood loss from these highly vascular tumors a risk factor for hemodynamic instability or cardiac arrest, including hyperkalemia associated with rapid transfusions.

## How to avoid complications

An adequate surgical approach-typically combining extensive far-lateral and retrosigmoid approaches with C1 laminectomy-should be utilized to ensure adequate exposure of the fourth ventricle, CPA, upper cervical spinal cord and clival region. IOM provides real-time warning against excessive CN manipulation [4].

Dissection near the cranial nerves should proceed in a lateral-to-medial direction, following the nerves from their exiting foramina toward the brainstem. Low-amplitude suction is preferable to the ultrasonic aspirator when working directly around fragile, encased nerves. Careful preservation of arachnoid planes is essential [4].

Local application of papaverine is recommended to improve local perfusion.

To prevent diagnostic uncertainty on postoperative MRI, all hemostatic agents should be removed from the resection cavity [4].

Lastly, recognizing the limits of safe dissection and planning early second-look surgery for residual tumor may represent a safer alternative to accepting excessive risk during the primary procedure.

## Specific information for the patient and family

Although children with lateral-type ependymomas often present with minor neurological deficits, aggressive surgical resection remains the standard of care. This necessary treatment strategy may be associated with a challenging postoperative course. Families should be informed about the potential occurrence of postoperative vocal cord paralysis, facial weakness, and swallowing dysfunction. As a result of these complications, children may require supportive interventions, including temporary tracheostomy or a gastrostomy tube [2, 3, 5]. The importance of achieving GTR, even at the cost of transient or minor permanent deficits, should be clearly emphasized during preoperative counseling [1, 4].

## Key points summary

1. Lateral-type ependymomas originate from the foramen of Luschka or the lateral aspect of the brainstem. As they grow, they expand the foramen of Luschka and extend into the fourth ventricle and CPA, frequently encasing important neurovascular structures.
2. The surgical objective should be GTR whenever safely achievable, as it represents the primary modifiable determinant of long-term survival.
3. Optimal exposure typically requires an extensive craniotomy combining a far-lateral and retrosigmoid approach with C1 laminectomy.
4. During tumor dissection from the cranial nerves, a lateral-to-medial trajectory should be followed to reduce the risk of permanent nerve avulsion.
5. Preservation of arachnoid planes is critical.
6. Tumor folds often contain a cranial nerve or a vessel at their base and should therefore be dissected with particular caution.
7. Local papaverine irrigation may improve regional perfusion and help reduce the risk of postoperative cranial nerve deficits.
8. If postoperative MRI reveals a residual tumor, second-look surgery should be strongly considered prior to adjuvant conformal radiotherapy.
9. A multidisciplinary approach, including pediatric oncology consultation, is essential for optimal patient outcomes.
10. Aggressive resection frequently causes temporary neurological deficits, specifically vocal cord paralysis and swallowing dysfunction. Most of these deficits, however, improve within months with appropriate supportive care.

## Supplementary Information

Below is the link to the electronic supplementary material.

ESM 1 Supplementary Material 1 (MP4 149 MB)

## Data Availability

No datasets were generated or analysed during the current study.
